# Supplementary material for: Dissecting the Determinants of Domain Insertion Tolerance and Allostery in Proteins
Source: Adv Sci (Weinh). 2023 Aug 10;10(28):2303496. doi: 10.1002/advs.202303496 (PMC10558690; doi:10.1002/advs.202303496)
Supplement: Supplementary file 1 — Supporting Information [file ADVS-10-2303496-s001.pdf]

ADVANCED SCIENCE
Open Access

Supporting Information

for *Adv. Sci.*, DOI 10.1002/advs.202303496

Dissecting the Determinants of Domain Insertion Tolerance and Allostery in Proteins

Jan Mathony, Sabine Aschenbrenner, Philipp Becker and Dominik Niopek**

Supporting Information

Dissecting the Determinants of Domain Insertion Tolerance and Allostery in Proteins

Jan Mathony, Sabine Aschenbrenner, Philipp Becker, Dominik Niopek**

Contents:

Text S1 to S2

Figs. S1 to S21

Tables S1 to S5

Supplementary References (#1 to #30)

Supplementary Text

Note S1. Analyzing Alphafold2 structure predictions of domain insertion variants.

In light of the recent advances in protein structure prediction, a logical question was if AF2 could guide the identification of promising domain fusions. We chose the pLDDT metric as a starting point, as it was previously shown to be correlated with flexible protein regions[1–3], and could hence serve as potential indicator for suitable domain insertion sites. Analysis of the pLDDT scores of individual amino acids from an AF2-derived structure of wildtype AraC revealed a trend towards lower pLDDT values at enriched sites, although the resulting correlation was very weak (Spearman's r of -0.26; Figure S13).

Next, we predicted AF2 structures of all possible PDZ insertions into AraC (Figure S14A). Representing all amino acid-wise pLDDT scores corresponding to AraC from each fusion protein in a heatmap allowed us to investigate the effect each insertion has on the pLDDT scores of AraC (Figure S14B). Most prominent in the resulting representation is a diagonal of decreased pLDDT values corresponding to the residues neighboring the respective position of the PDZ insertion. These lower values could implicate structural flexibility around the respective insertion site. The interpretation is backed by the fact that the unstructured loops of AraC are also visible as vertical regions with decreased pLDDT scores. We note that the structure of the N-terminal β-barrel (AA 20-100) is implicitly visible in the heatmap by a symmetric pattern of locally decreased pLDDT scores indicating its loop regions in the upper left quarter. Indeed, the pLDDT scores reflected structural features of AraC and potentially local conformational effects of insertions, albeit these findings remain speculative as this point. However, the pLDDT score changes did not correlate with the experimentally determined enrichment scores (Figure S14C).

In line with the pLDDT values, the structural differences between predicted models of wildtype AraC and the corresponding parts of AraC-PDZ hybrid structures exhibited a similar trend (Figure S14D). When, in turn, the PDZ insert was compared to its wildtype conformation (Figure S14E), misfolding of the domain was predicted for several hybrids, although the corresponding insertion sites did not necessarily correspond to regions of significant depletion in our screen. Taken together, the exploration of predicted hybrid protein structures suggested that AF2 is not able to capture the functional effects of domain insertions in a meaningful way. Given the generally lower performance of AF2 on multi-domain proteins[4], domain insertion engineering might still

be beyond the scope of AF2 and similar state-of-the-art structure prediction methods. Nonetheless, AF2 predictions do reflect diverse structural features of AraC.

Note S2. Optogenetic AraC variants and single-protein Boolean logic gates.

Boolean logic computations are typical elements of genetic circuits and programs used in synthetic biology. They are usually implemented at the transcriptional and/or translational level[5,6], which causes delays in the signal relay and integration. Protein-based logic computation in contrast does not suffer from these limitations and thus holds great potential for the custom control of cellular processes and the implementation of computational circuits in cells.[7,8] However, increasingly complex circuit designs require the use of a large number of individual protein components as well as their efficient communication via protein-protein interactions.[7,8] A recent preprint, for instance, impressively demonstrated design of neural-network computations on the protein level[9], showcasing the increasing power of artificial protein networks in living cells. Such complex cellular compute programs, however, show considerable noise and potentially cross-talk within the system and thus require efficient processing at the level of the individual protein components. In contrast to such commonly used protein logic gate designs based on several separate protein components, our AraC-LOV2 fusions represent single protein Boolean logic gates (Figure 4B). AraC-I113-LOV2 acts as an AND-gate, integrating blue light and arabinose as inputs, while AraC-S170-LOV2 represents a NIMPLY-gate. We have found only two other examples of engineered, single-protein logic gates in the literature. The first was constructed by fusing LOV2 and uniRapR domains to a kinase[10] resulting in an OR gate behavior, while the second comprises an engineered transcription factor that responds to light and temperature changes.[11] A particular advantage of these single protein-based logic gates is that they provide a direct wiring from the input signals to the output computation within a single polypeptide chain, a computation that would otherwise require several separate protein components. Building on this unique feature could highly simplify the design of compute circuits and their operation in living cells. Our combination of the naturally existing allosteric signaling in AraC with an artificial, second input might be an engineering approach that could be easily adapted to other proteins and thereby facilitate the implementation of Boolean logics for demanding computational operations in cells.

Taken together, the possibility to integrate complex information and wiring it to desired actuations is one of the main goals in synthetic biology. Integrating more functions into single amino acid chains therefore has the potential to simplify molecular networks, release metabolic burden from the host cell[12] and reduce noise derived from stochastic fluctuations of the individual components.[13] This way, single-protein logic gates could contribute to future generations of synthetic biology approaches to program and re-wire cells.

Supplementary Figures and Figure Legends

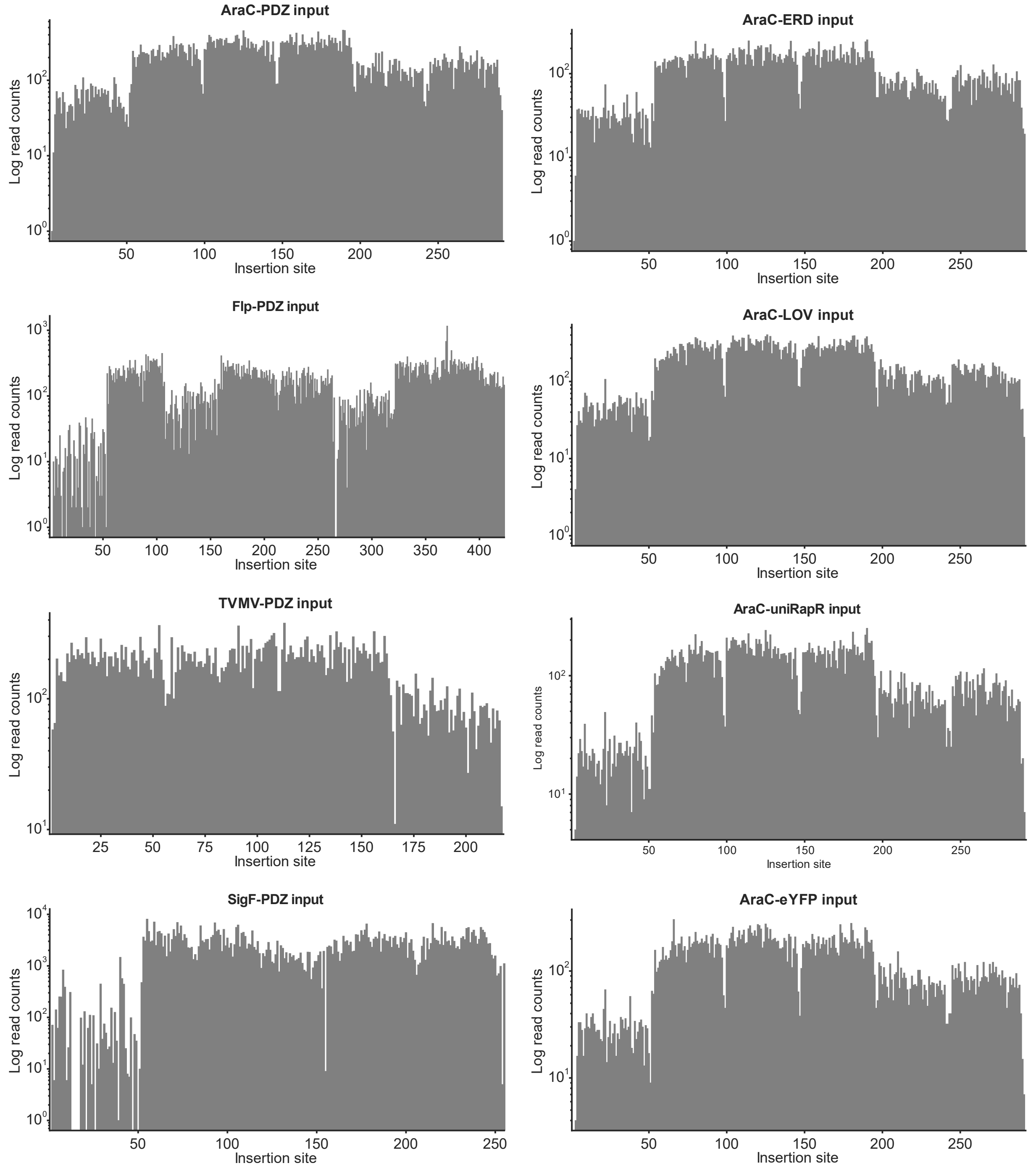

Figure S1. Cloning of domain insertion libraries via SPINE yields near-complete coverage of domain insertion positions.

The insertion library coverage was assessed via NGS. Histograms represent the log-normalized read counts for insertions at the respective position (amino acid/codon).

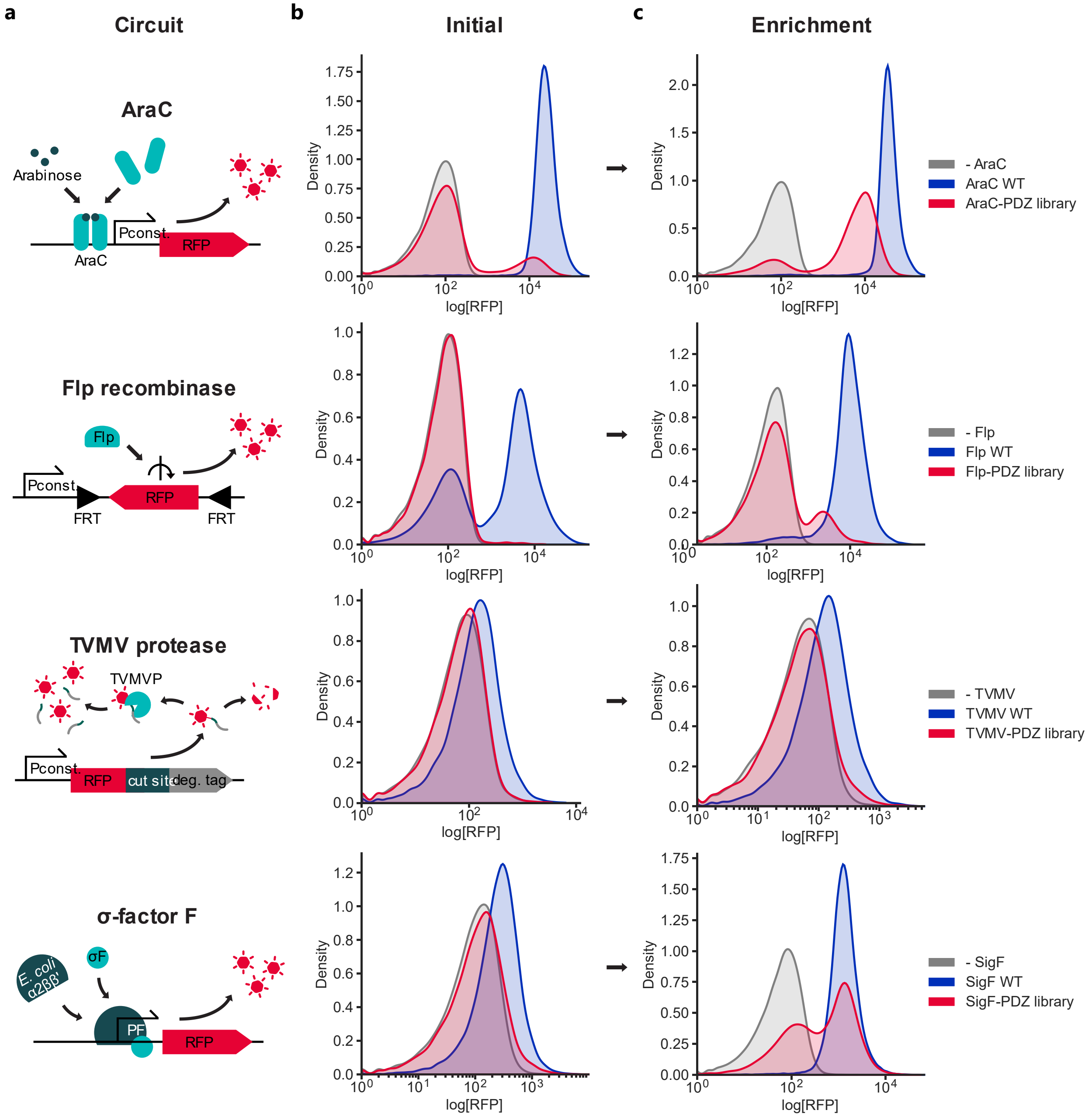

Figure S2. Enrichment of active effector-insert hybrids from candidate libraries via FACS. (A) Schematics of the reporter assays for AraC, the Flp recombinase, the TVMV protease and SigF are shown. (B, C) Histograms depicting the RFP fluorescence distribution during FACS-based library enrichment. Representative histograms generated from 25,000 gated events of (B) the initial library and (C) after the first enrichment are shown. The negative controls (-) carried a plasmid expressing a different candidate protein not activating the reporter construct.

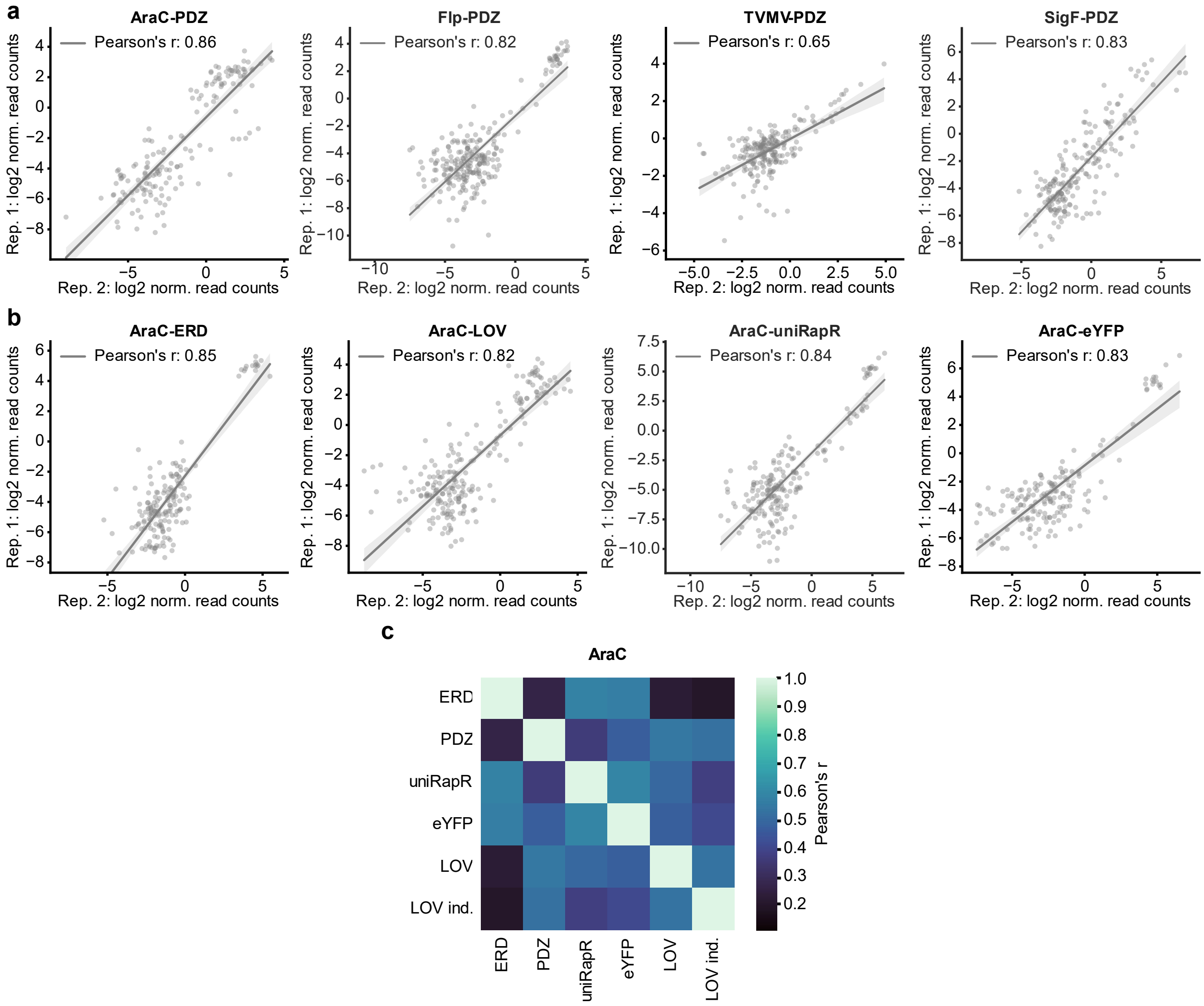

Figure S3. Domain insertion profiling outcomes are highly reproducible.

(A, B) The enrichment scores of biological replicate-1 are plotted against the respective scores from a second replicate-2 for the different effector-PDZ libraries (A) and the additional AraC libraries with varying insert domains (B). Only variants that were not fully depleted during enrichment are shown. A linear fit with 95 % confidence intervals is included and Pearson correlations coefficients are indicated. Rep., replicate; norm., normalized. c, The heatmap shows pairwise Pearson correlations between all domain inserted into AraC. Enrichments of the AraC-LOV2 library in darkness and under light induction (ind.) were assessed and are depicted separately.

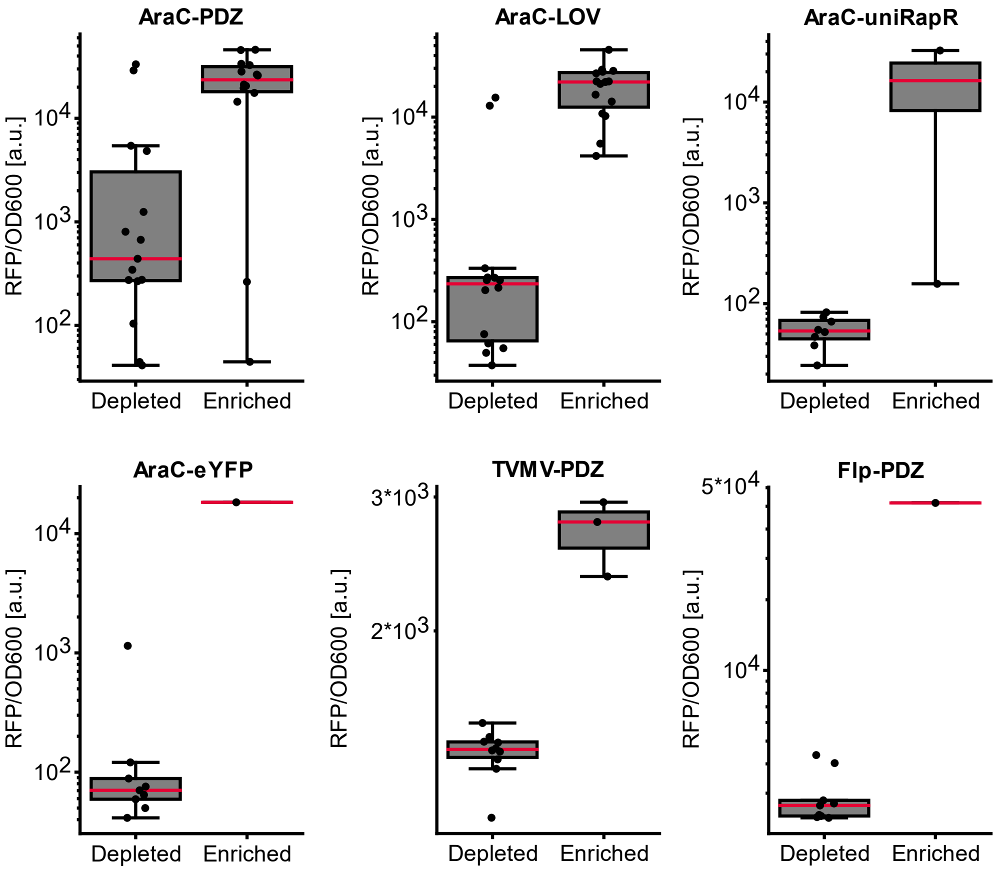

Figure S4. Cross-validation of the domain insertion screen by experimental characterization of individual insertion variants.

Individual domain insertion variants were cloned and their activity was assessed using the respective RFP reporter assays. Boxplots indicate the resulting normalized fluorescence for enriched and depleted candidate. Individual data points correspond to the mean of three biological replicates, each of which reflect of three underlying technical replicates. The IQR is marked by the box and the median is represented by a red line. Whiskers extend to the 1.5-fold IQR or to the value of the smallest or largest enrichment, respectively.

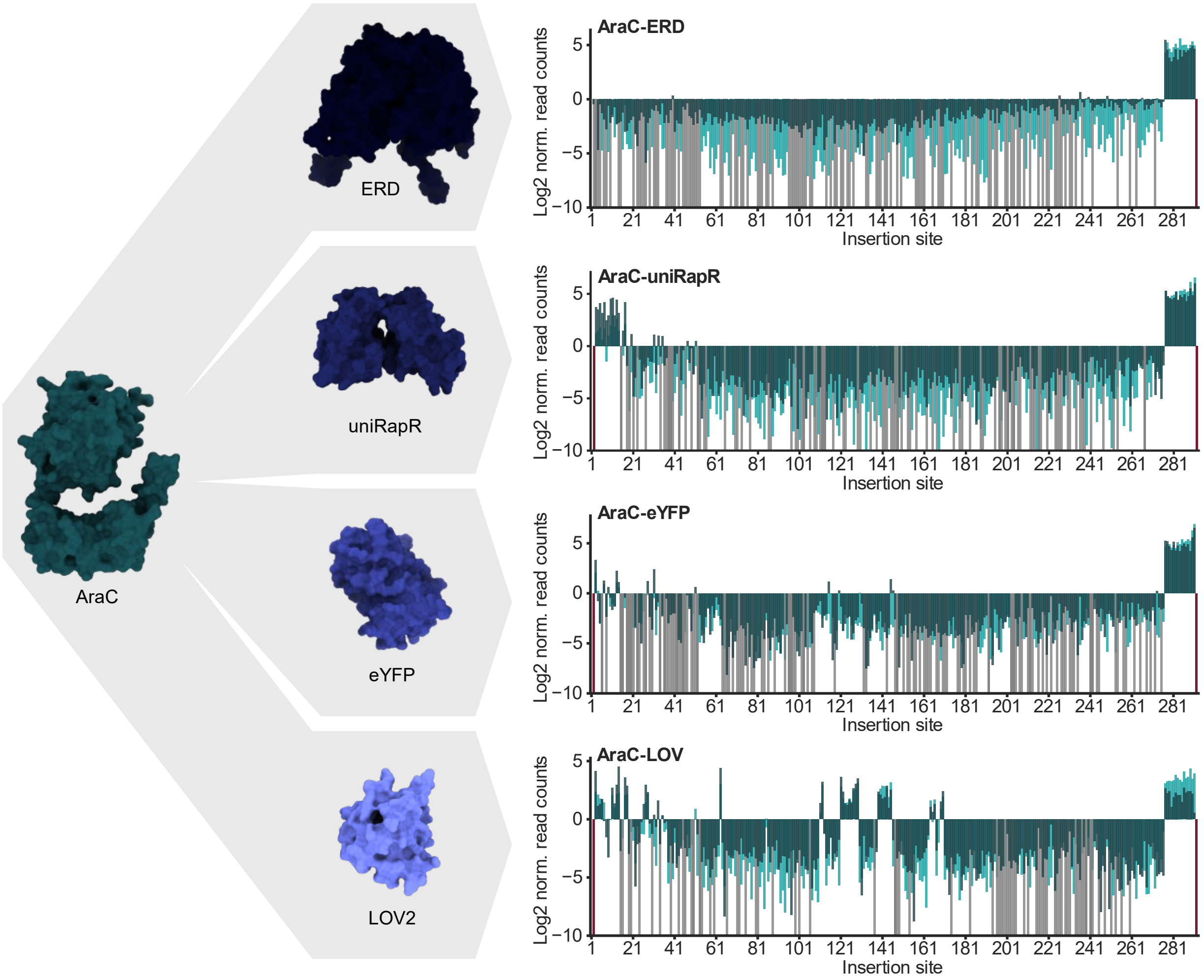

Figure S5. Domain insertion tolerance depends on the identity of the insert.

Results from insertion screens of AraC with the ERD, LOV2, uniRapR and eYFP insert domains are shown. Enrichments are mapped to the respective insertion site as indicated by the position of the AraC preceding the insertion. Light green, dark green: individual replicates. Grey: variants with zero reads after enrichment. Red: variants missing in the initial library.

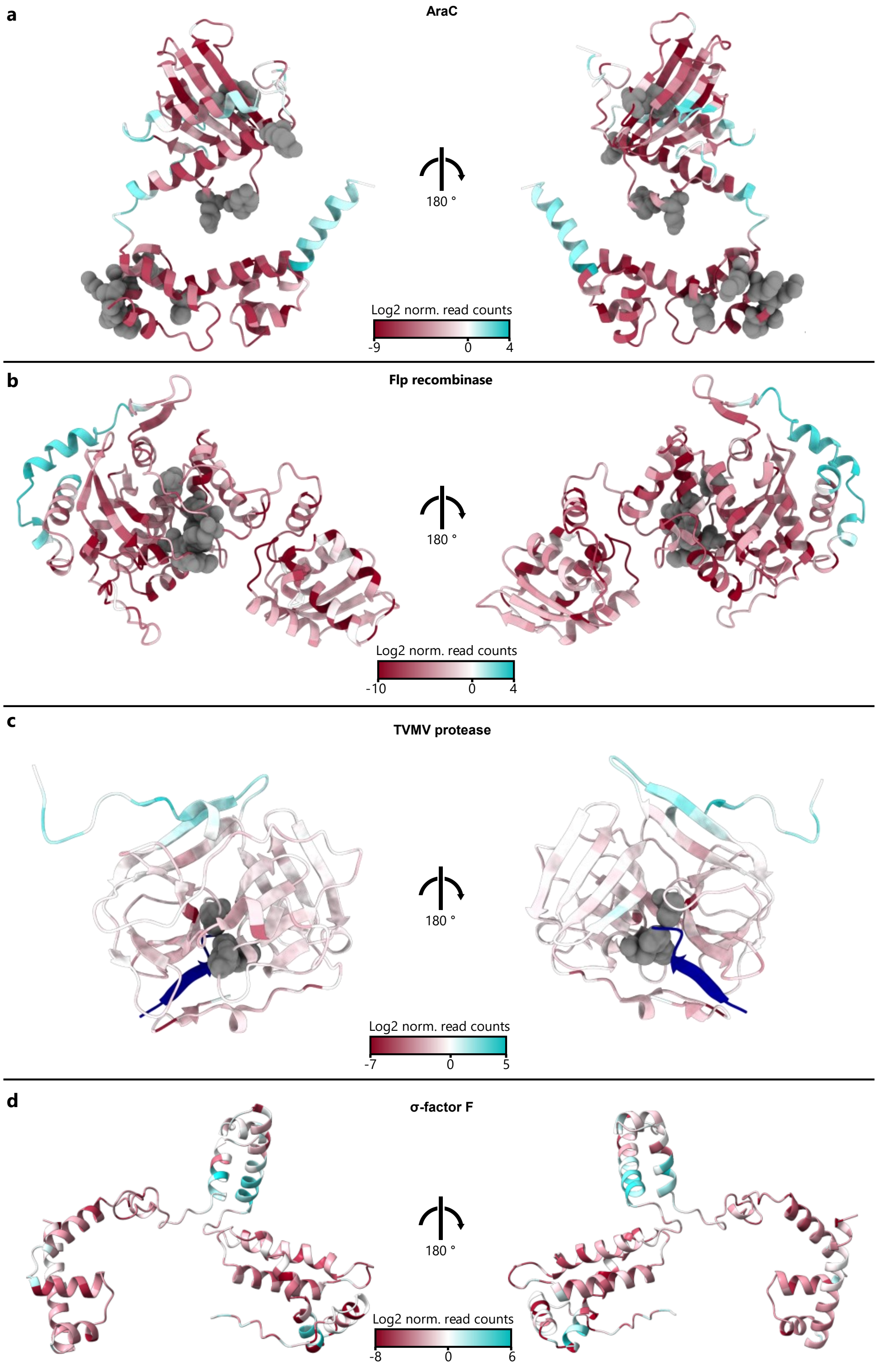

Figure S6. Positions with insertion tolerance are clustered at distinct, locally confined surface sites.

(A-D), The insertion scores from the PDZ libraries are mapped onto the AF2 structure predictions of AraC (A), and the Flp recombinase (B), a crystal structure of the TVMV protease (PDB-ID: 3MMG) (C) and an AF2 structure prediction of SigF (D). In C, the TVMV protease substrate is depicted in blue. Functionally critical residues are shown in grey for AraC, Flp and the TVMV protease.

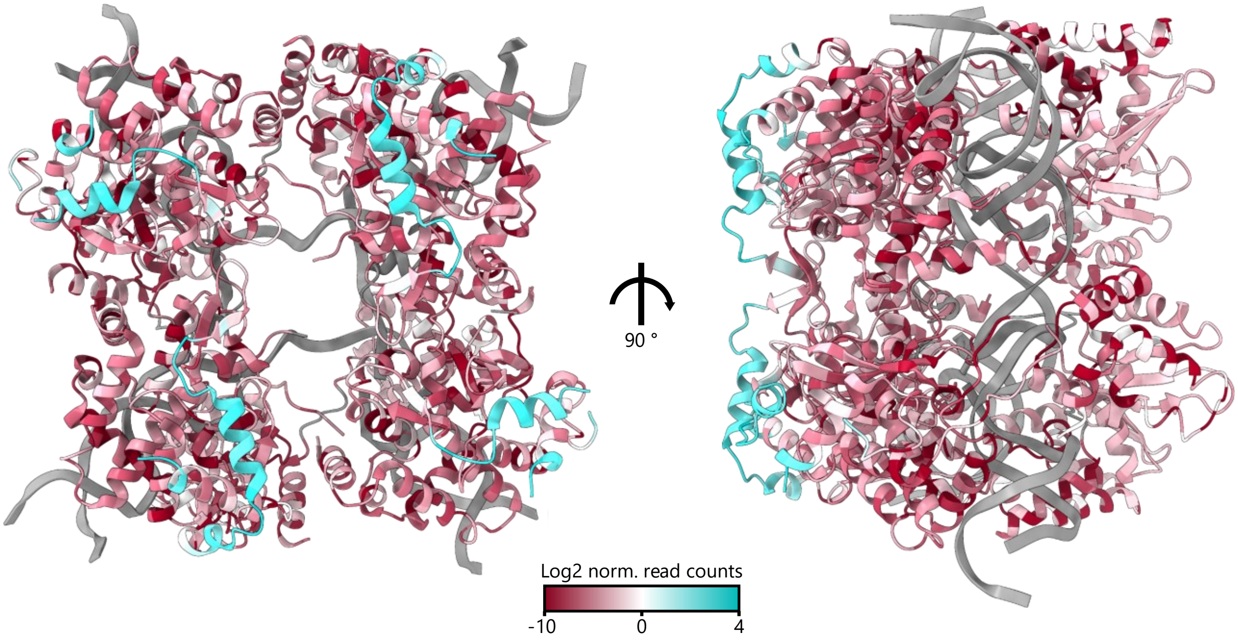

Figure S7. Enrichment scores mapped onto structures of the Flp-holliday junction complex. PDB-ID: 1FLO.

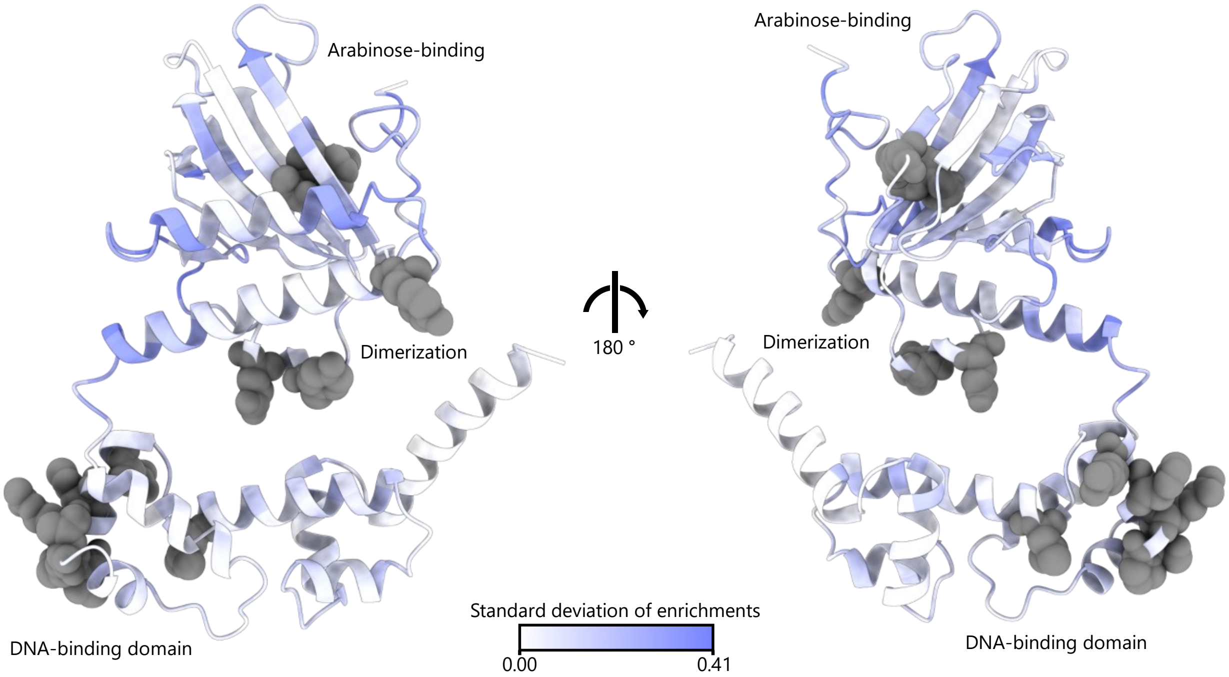

Figure S8. Insertion permissive regions are scattered across AraC and depend on the insert domain.

The AF2-derived structure of AraC is colored by the SD of the min-max-scaled enrichment scores from all insert libraries corresponding to five different insertion domains. Functionally critical residues are highlighted in grey.

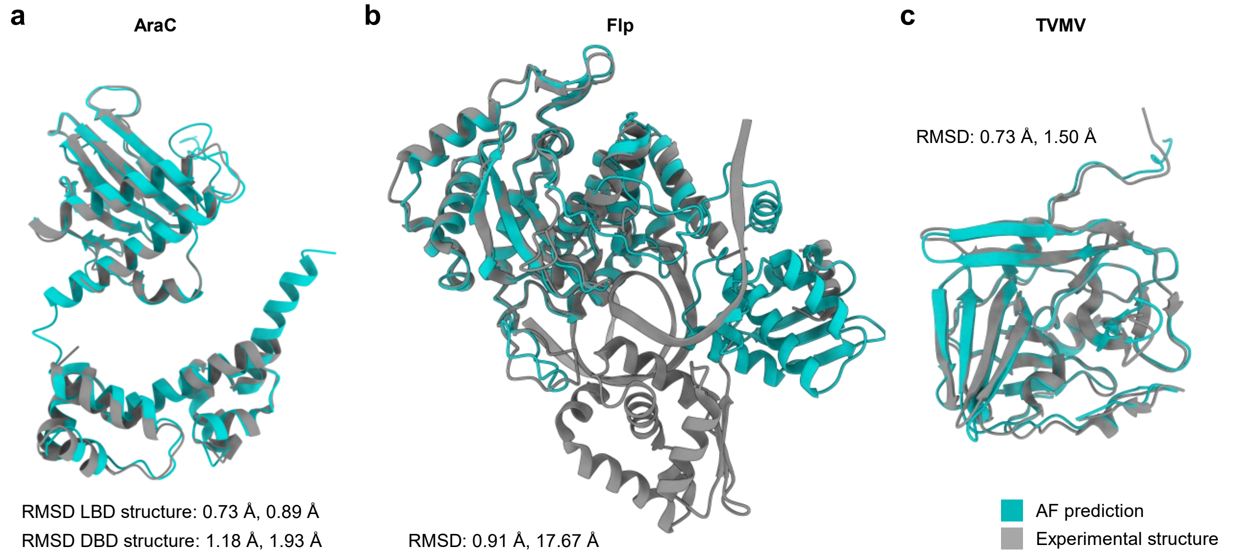

Figure S9. AlphaFold2 predictions accurately capture the structures of the candidate proteins.

(A-C), Structural alignments between experimentally resolved structures (grey) and AlphaFold2 predictions (green) are shown for AraC (A), Flp (B) and the TVMV protease (C). The RMSD of the aligned residues as well as the RMSD for all amino acids are shown. PDB-IDs: 2ARA, 2K9S, 1FLO, 3MMG.

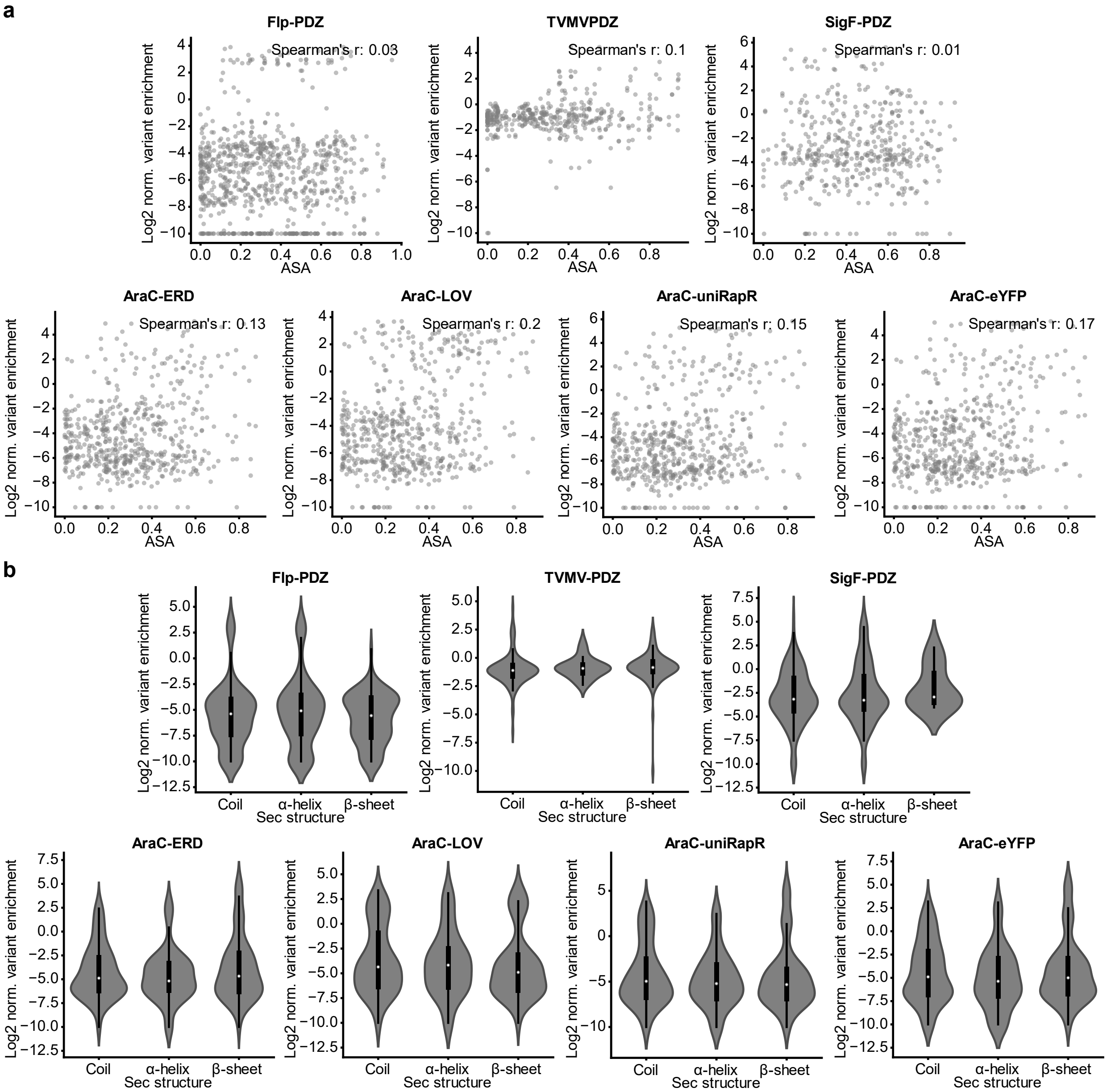

Figure S10. Correlations between the enrichment scores and surface accessibility or secondary structures.

(A) Scatter plot showing the relation between variant enrichment and the average surface exposed area (ASA) of the residues neighboring an insertion site. (B) The insertion score in regions with the respective secondary structure element are shown. For each insertion site, the secondary structure assignment of the amino acid prior and after the insertion were considered. The IQR is

marked by the box and the median is represented by the white dot. Whiskers extend to the 1.5-fold IQR or to the value of the smallest or largest enrichment, respectively.

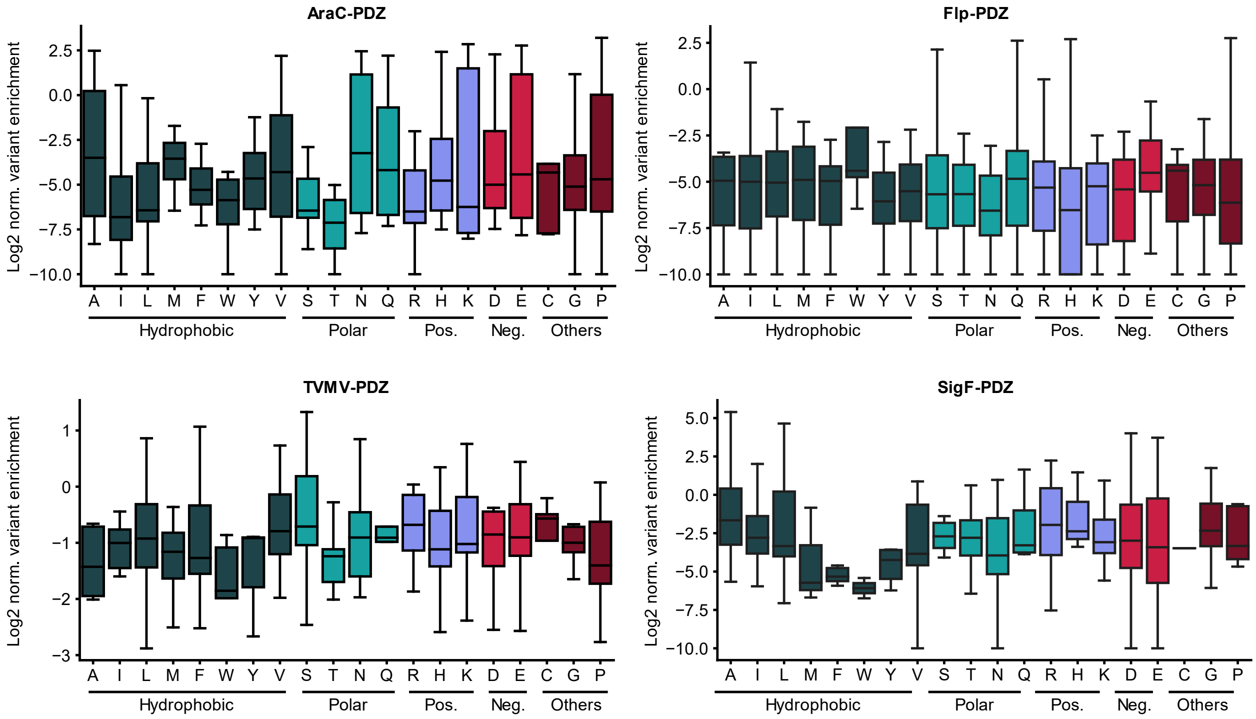

Figure S11. Successful domain insertion cannot be predicted from amino acid identity. (A-D) The enrichment score distribution for each amino acid is shown as boxplots for the PDZ libraries of AraC (A), Flp (B), TVMV protease (C) and SigF (D). Both residues neighboring an insertion site were taken into account for the calculations. The IQR is marked by the box and the median is represented by a line within the box. Whiskers extend to the 1.5-fold interquartile range (IQR) or to the value of the smallest or largest enrichment. Colors indicate the different amino acid categories as indicated underneath the plots. Pos., positive charged. Neg., negatively charged.

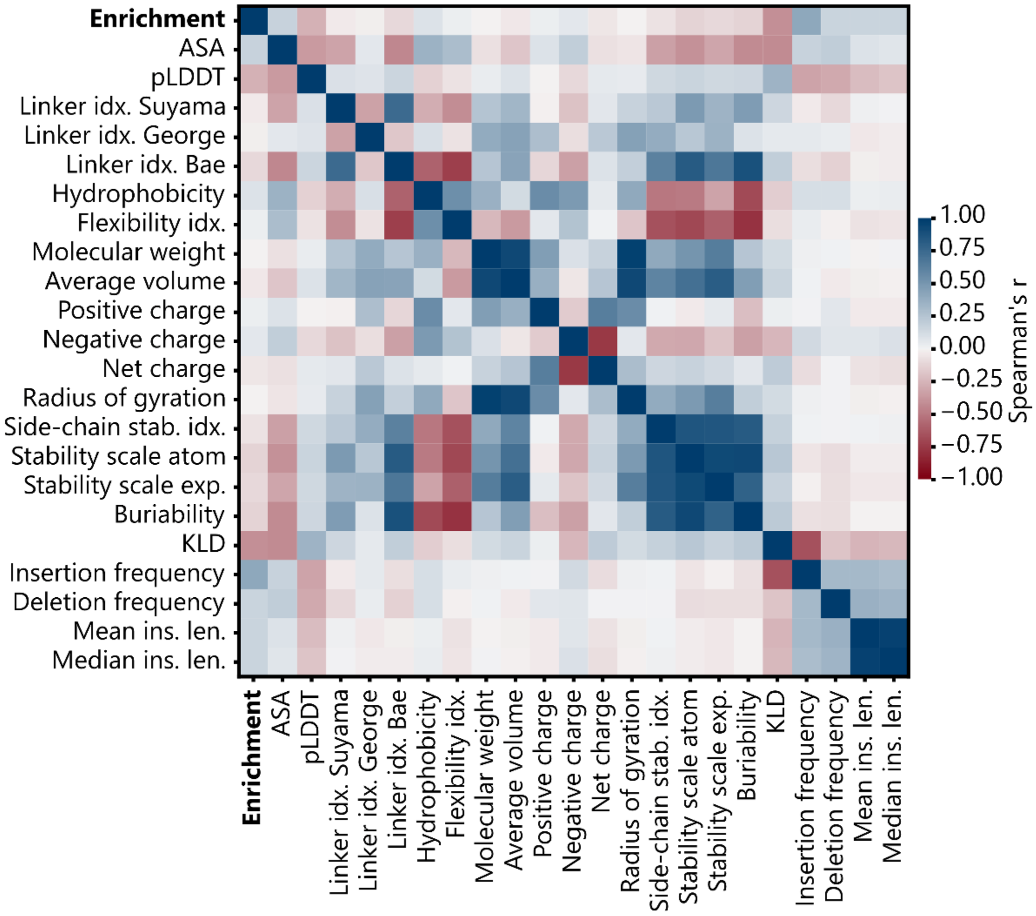

Figure S12. Heatmap of pairwise Spearman correlations between all investigated positional features.

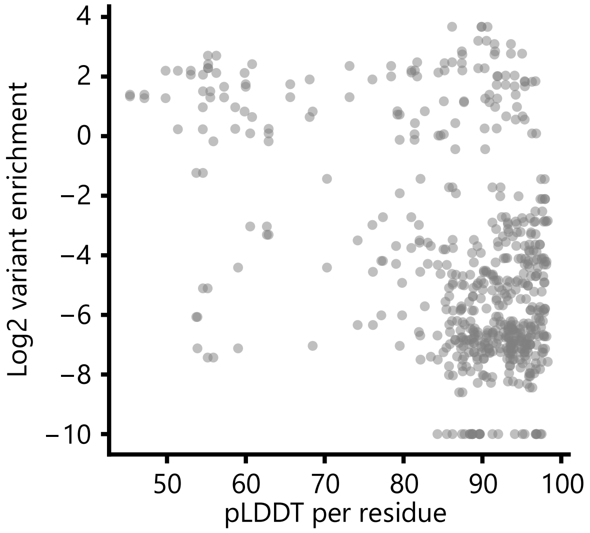

Figure S13. The position-specific pLDDT scores of wildtype AraC do not correlate with domain insertion susceptibility.

Scatterplot of the relation between the enrichment scores of the AraC-PDZ library and the amino acid pLDDT scores from an AraC structure predicted by AF2. The corresponding Spearman's r is -0.26.

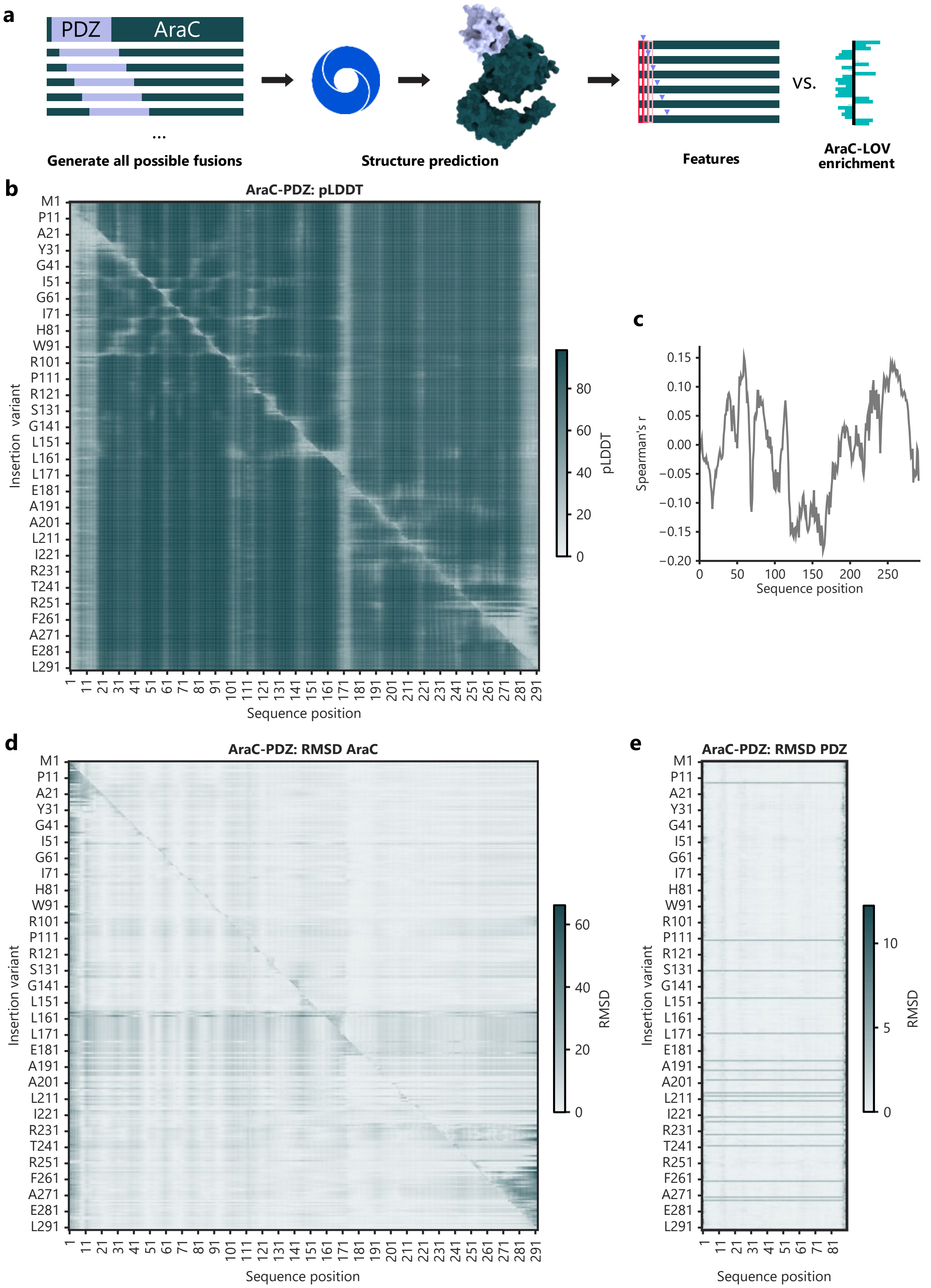

Figure S14. Correlations of AF2 structure predictions with domain insertion susceptibility.

(A) Depiction of the structure prediction workflow. Structures for all possible insertions of the PDZ domain into AraC were generated with AF2. Structural changes at single positions in response to different insertions were then compared and correlated to the experimental enrichments. (B) Structures of all possible PDZ insertions into AraC were predicted. The heatmap shows the pLDDT scores per position for each variant. Only AraC amino acids are depicted so that each column corresponds to pLDDT values from the same residue in different insertion variants. Rows, in turn, correspond to the different AraC-PDZ hybrids. (C) For each amino acid position, the pLDDT scores from all variants (columns in B) were correlated with the corresponding enrichment scores at these positions. The resulting Spearman correlation coefficients are shown. (D-E) The predicted AraC-PDZ structures were aligned to a predicted structure of wildtype AraC (D) or PDZ ©. The RMSDs between the wildtype and the respective part of the hybrid proteins are shown in the heatmap. Rows correspond to the different AraC-PDZ hybrids and columns to RMSD values of the same residue in different variants.

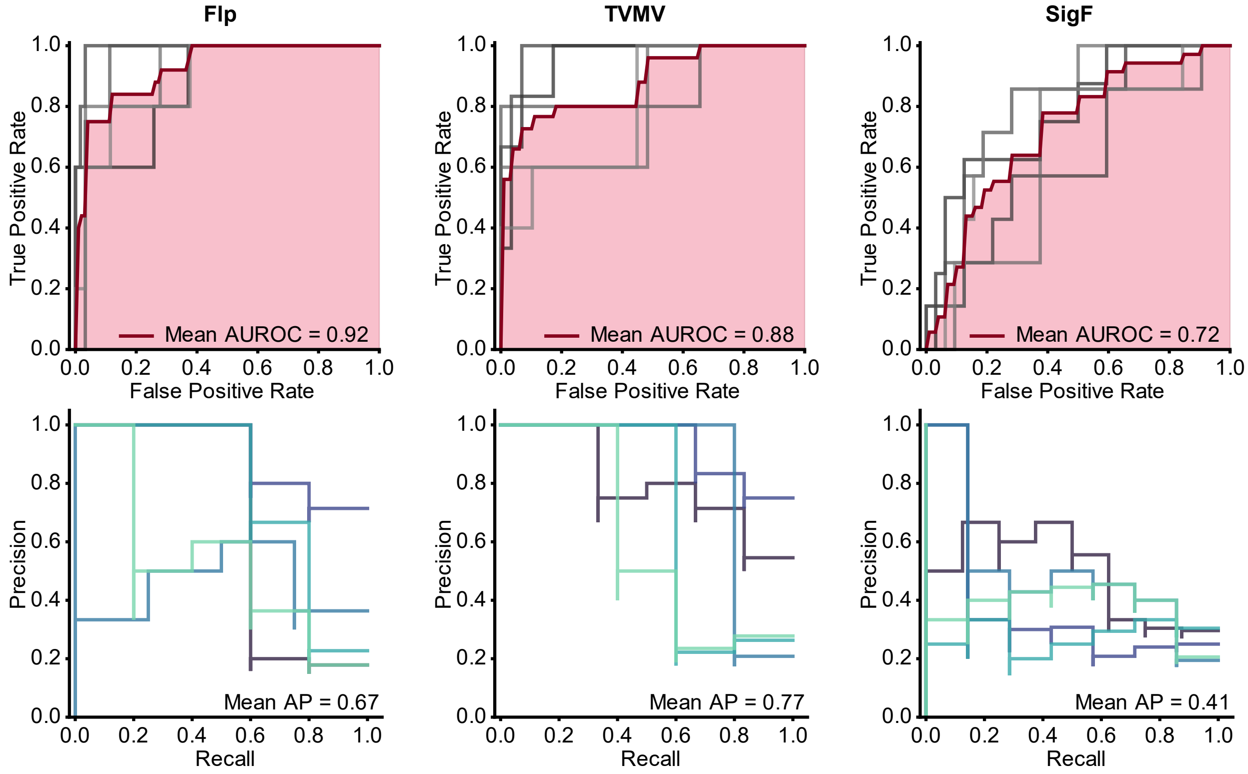

Figure S15. Gradient boosting models trained on positional features can infer insertion tolerance for individual proteins.

Performance metrics of gradient boosting classifiers that were trained on the PDZ datasets for Flp, TVMV protease and SigF with five-fold cross-validation are shown. The ROC (top) and precision-recall curves (bottom) are depicted for individual folds. The mean ROC is shown in red and the mean AUC is marked in light red.

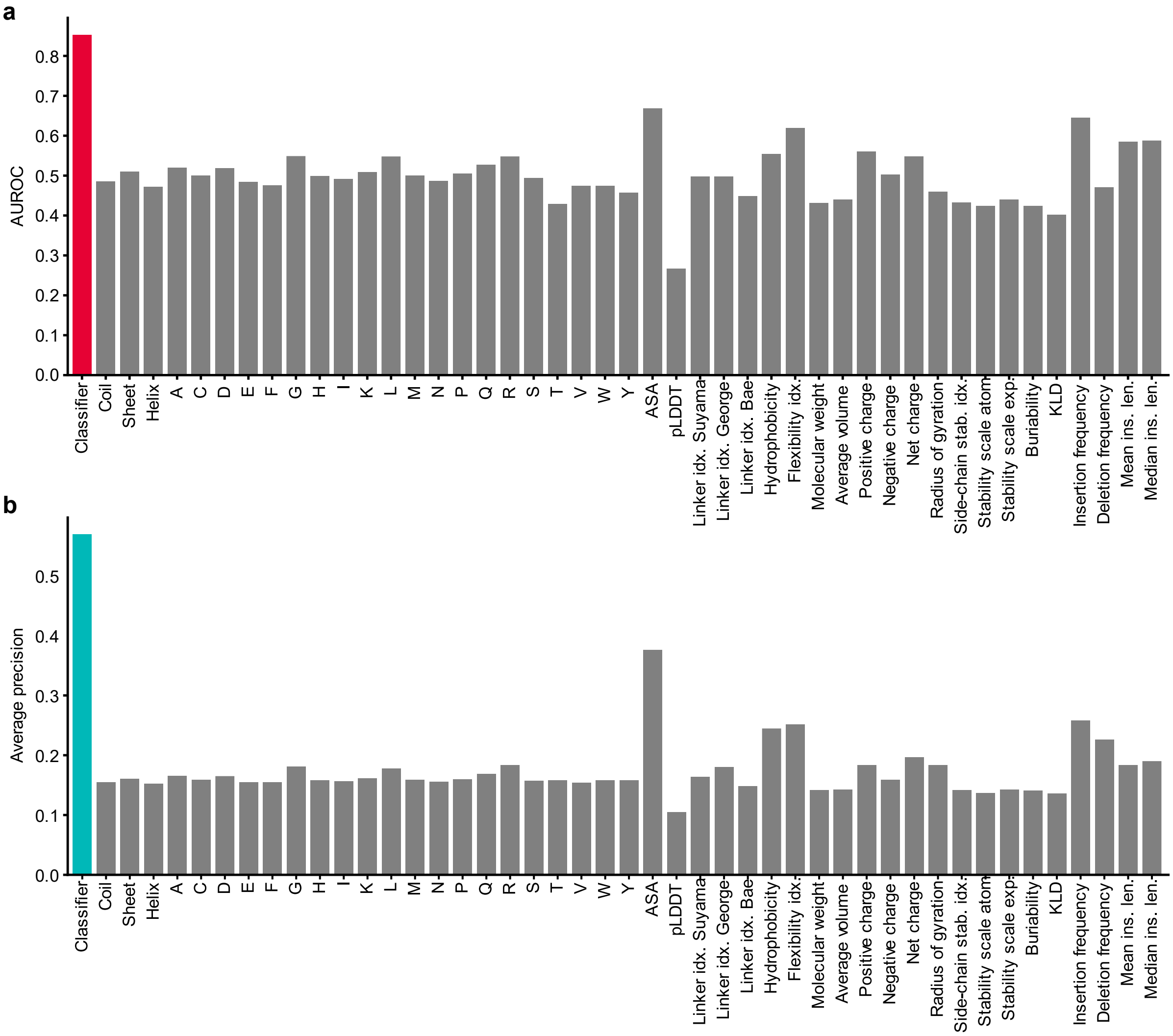

Figure S16. Full comparison of the trained classifier to baseline predictors.

(A-B) The mean AUROC (A) and average precision (B) are shown. The values were calculated on a previously withheld test set. The performance of the gradient boosting classifier is compared to all individual features.

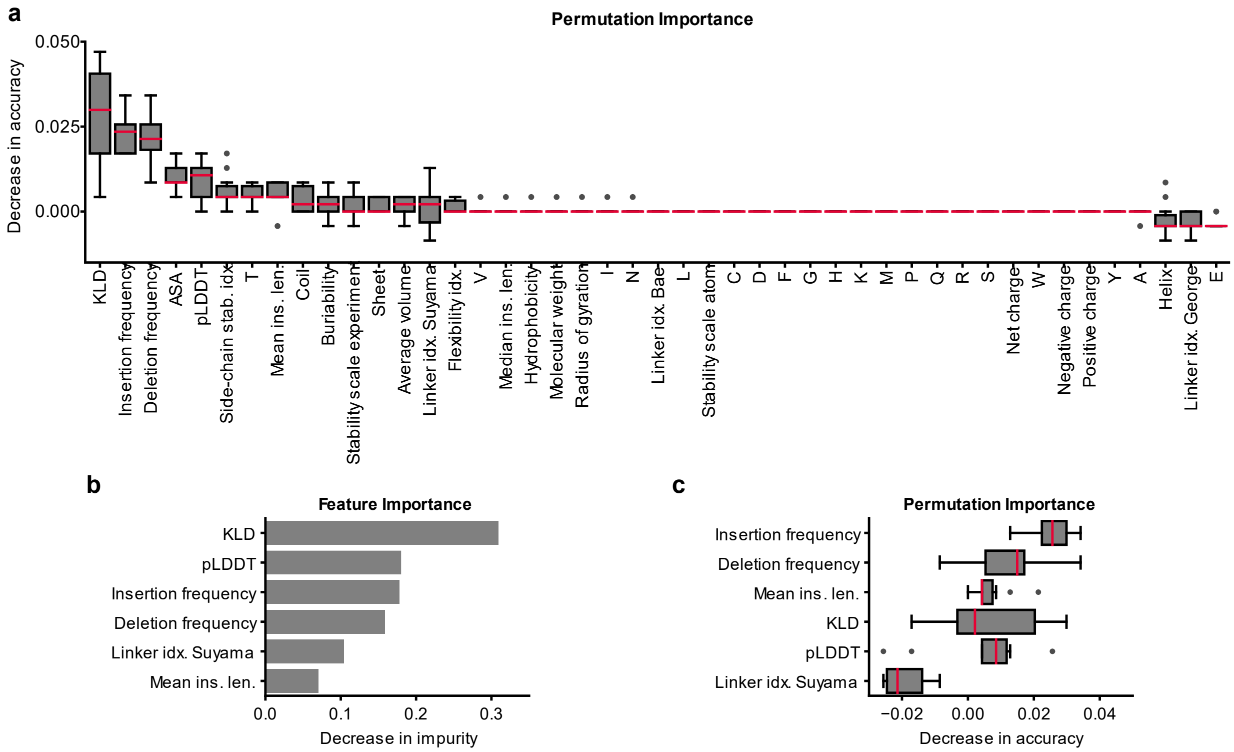

Figure S17 | Alignment-derived statistics are key predictors of insertion tolerance.
(A) The decrease in accuracy upon random permutation of the respective features is presented for the gradient boosting model trained on the complete dataset. (B) Bar plot indicating the Gini importance of each feature of the reduced model. (C) The permutation importance of training features of the reduced model is shown. (A, C) The results were calculated individually for each structure in the cross-validation dataset. The IQR is marked by the box and the median is represented by a red line. Whiskers extend to the 1.5-fold IQR or to the value of the smallest or largest score, respectively. Outliers are shown as points.

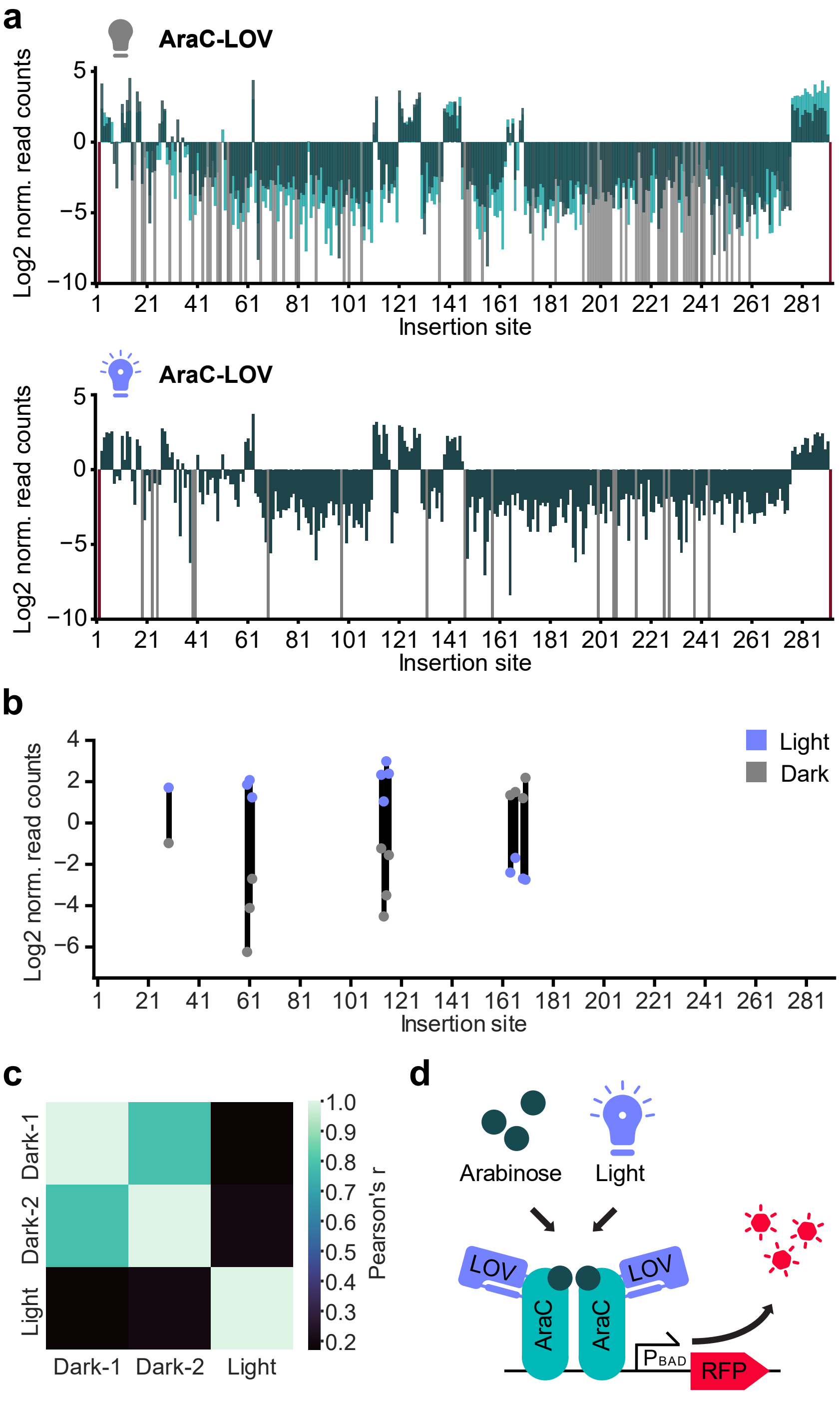

Figure S18. Distribution of light-switchable variants in the AraC-LOV2 dataset.

(A) Enrichment scores of AraC-LOV libraries that were sorted following incubation in darkness (upper panel) or under blue-light exposure (lower panel) are mapped onto the corresponding insertion sites of AraC (preceding the indicated residue). Values for the light exposed sample correspond to a single experiment. For the sample incubated in the dark, light green and dark green

indicates individual replicates. Grey: variants with zero reads after enrichment. Red: variants missing in the initial library. (B) Enrichment scores derived from experiments under light exposure or in darkness are marked by blue and grey points, respectively. Only datapoints from promising candidates with a log2 enrichment >1 in the active state and a log2 difference >2.5 between the light and dark states are shown. (C) Pearson correlations between the different datasets are shown. Only positions of interest, that exhibited an enrichment in at least one replicate were included in the calculation. (D) Schematic of the co-dependence of the AraC-LOV2 hybrids on arabinose and blue light.

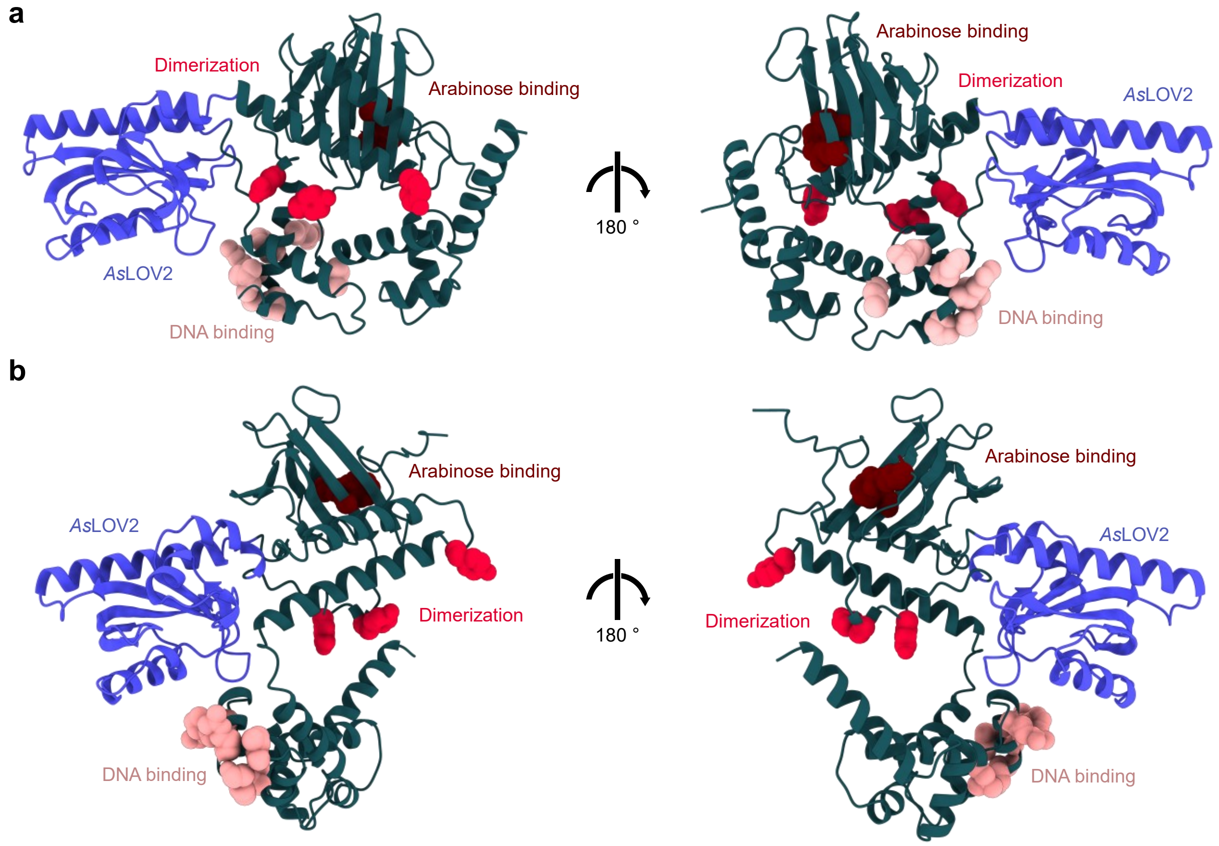

Figure S19. AlphaFold2 predicts different conformations for the lead AraC insertion variants.

(A, B) AF2 predictions of AraC-I113-LOV2 (A) and AraC-S170-LOV2 (B) are shown. AraC is depicted in green and the *As*LOV2 domain in blue. Residues that bind to the operator are highlighted in pink, key residues for dimerization in the induced state in red and the amino acids that are important for arabinose binding in vermilion.

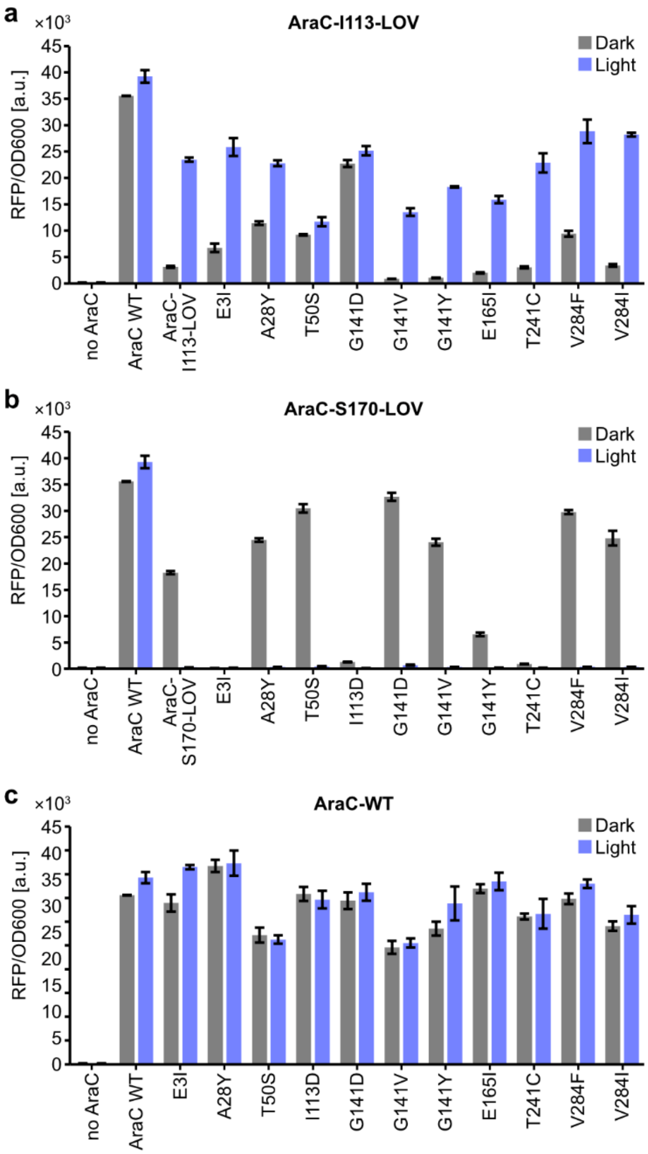

Figure S20. Point mutations improve the performance of the AraC-S170-LOV light switch. (A-C) Cultures were inoculated from precultures carrying plasmids encoding an RFP reporter and the indicated AraC-I113-LOV (A), AraC-S170-LOV (B) and AraC (C) point mutants. The samples were incubated for 16 h under light exposure or in darkness at an arabinose concentration of 8 mM, followed by plate reader measurements of RFP fluorescence and OD600. Bars represent means from three independent biological replicates. Error bars show the SD.

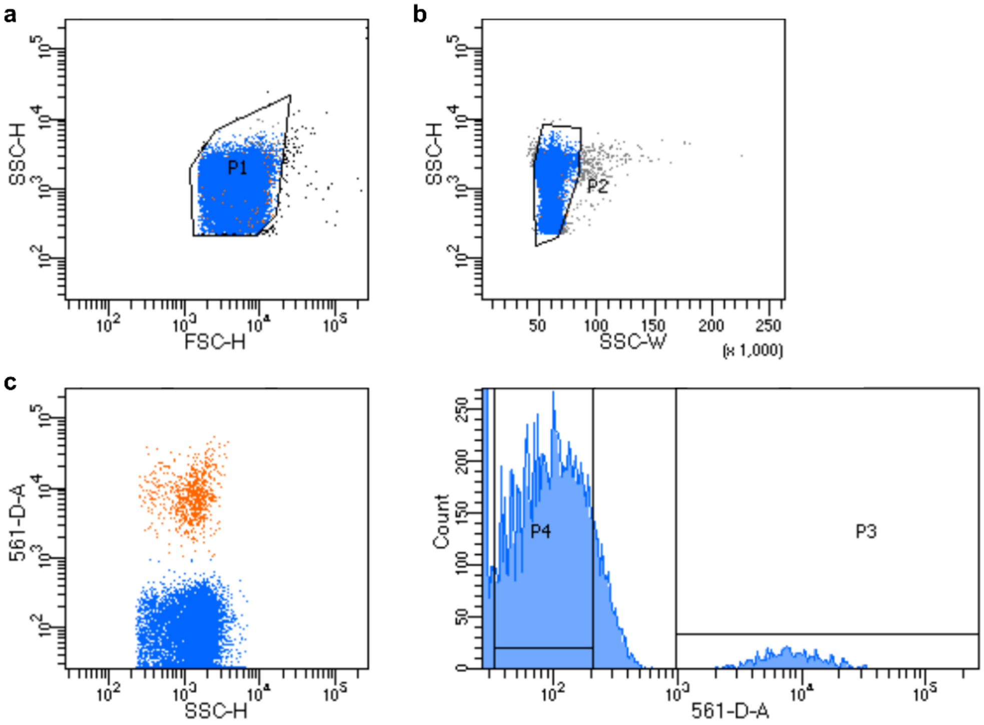

Figure S21. Gating strategy used during sorting.

(A) Scatter plot indicating how cells were selected via their forward and side scatter. (B) Scatter plot of side scatter height and width showing the gate that was set for the selection of singlets. (C) The population of red fluorescent bacteria was sorted as indicated in the scatter plot and the histogram of the measured RFP fluorescence.

Table S1. Molecular properties of the insert domains.

Insert domain	Sequence length	Mol. weight	Distance of termini
*As*LOV2	141 AAs	16.3 kDa	20.7 Å
ER domain	257 AAs	29.2 kDa	63.8 Å
eYFP	238 AAs	26.9 kDa	28.3 Å
PDZ domain	86 AAs	9.2 kDa	14.1 Å
uniRapR	198 AAs	22.1 kDa	24.4 Å

Table S2. Position-specific properties used for the analysis of domain insertion tolerance.

Property	Description
ASA	Average surface accessibility
pLDDT	Position-wise pLDDT of AF2 models
Linker idx. Suyama	Linker propensity index [14]
Linker idx. George	Linker propensity index [15]
Linker idx. Bae	Linker index [16]
Hydrophobicity	Hydrophobicity [17]
Flexibility idx.	Flexibility index [18]
Molecular weight	Molecular amino acid weight
Average volume	Average amino acid volume
Positive charge	Positive charged amino acid
Negative charge	Negative charged amino acid
Net charge	Net charge of amino acid
Radius of gyration	Radius of gyration of the side chain
Side-chain stab. Idx.	Side-chain contribution to protein stability (KJ/mol) [19]
Buriability	Buriability [20]
KLD	KLD Kullback-Leibler divergence calculated from MSA
Insertion frequency	Insertion frequency in related natural sequences at the respective position
Deletion frequency	Deletion frequency in related natural sequences at the respective position
Mean ins. len.	Mean insertion length in related natural sequences at the respective position
Median ins. len.	Median insertion length in related natural sequences at the respective position

Table S3. Constructs created and used in this study.

#	Name	Description. In sequential order
**1**	RFP reporter for AraC	BAD promoter, mRFP1, LVA degradation tag
**2**	RFP reporter for SigF	F1 promoter, mRFP1
**3**	RFP reporter for Flp recombinase	J23102 promoter, inverted mRFP1, flanked by FRT sites
**4**	RFP reporter for TVMV protease (J23105 + M0051)	J23105 promoter, mRFP1, TVMV recognition site, (M0051) DAS+2 degradation tag
**5**	AraC	TRC promoter, AraC
**6**	Flp recombinase	TRC promoter, Flp recombinase
**7**	TVMV protease	TRC promoter, TVMV protease
**8**	SigF	TRC promoter, SigF
**9**	AraC_S170_LOV2	TRC promoter, AraC with insertion of AsLOV2 behind S170
**10**	AraC_I113_LOV2	TRC promoter, AraC with insertion of AsLOV2 behind I113
**11**	AraC_E3_PDZ	AraC with insertion of PDZ behind E3
**12**	AraC_S14_PDZ	AraC with insertion of PDZ behind S14
**13**	AraC_N16_PDZ	AraC with insertion of PDZ behind N16
**14**	AraC_A17_PDZ	AraC with insertion of PDZ behind A17
**15**	AraC_L23_PDZ	AraC with insertion of PDZ behind L23
**16**	AraC_E27_PDZ	AraC with insertion of PDZ behind E27
**17**	AraC_T50_PDZ	AraC with insertion of PDZ behind T50
**18**	AraC_Q60_PDZ	AraC with insertion of PDZ behind Q60
**19**	AraC_E63_PDZ	AraC with insertion of PDZ behind E63
**20**	AraC_S112_PDZ	AraC with insertion of PDZ behind S112
**21**	AraC_I113_PDZ	AraC with insertion of PDZ behind I113
**22**	AraC_N116_PDZ	AraC with insertion of PDZ behind N116
**23**	AraC_R121_PDZ	AraC with insertion of PDZ behind R121
**24**	AraC_H129_PDZ	AraC with insertion of PDZ behind H129
**25**	AraC_G143_PDZ	AraC with insertion of PDZ behind G143
**26**	AraC_E165_PDZ	AraC with insertion of PDZ behind E165
**27**	AraC_S170_PDZ	AraC with insertion of PDZ behind S170
**28**	AraC_T241_PDZ	AraC with insertion of PDZ behind T241
**29**	AraC_D286_PDZ	AraC with insertion of PDZ behind D286
**30**	AraC_E3_LOV2	AraC with insertion of LOV2 behind E3
**31**	AraC_S14_LOV2	AraC with insertion of LOV2 behind S14
**32**	AraC_N16_LOV2	AraC with insertion of LOV2 behind N16
**33**	AraC_A17_LOV2	AraC with insertion of LOV2 behind A17
**34**	AraC_L23_LOV2	AraC with insertion of LOV2 behind L23
**35**	AraC_E27_LOV2	AraC with insertion of LOV2 behind E27
**36**	AraC_T50_LOV2	AraC with insertion of LOV2 behind T50
**37**	AraC_Q60_LOV2	AraC with insertion of LOV2 behind Q60
**38**	AraC_E63_LOV2	AraC with insertion of LOV2 behind E63
**39**	AraC_S112_LOV2	AraC with insertion of LOV2 behind S112
**40**	AraC_I113_LOV2	AraC with insertion of LOV2 behind I113
**41**	AraC_N116_LOV2	AraC with insertion of LOV2 behind N116
**42**	AraC_R121_LOV2	AraC with insertion of LOV2 behind R121
**43**	AraC_H129_LOV2	AraC with insertion of LOV2 behind H129
**44**	AraC_G143_LOV2	AraC with insertion of LOV2 behind G143
**45**	AraC_E165_LOV2	AraC with insertion of LOV2 behind E165
**46**	AraC_S170_LOV2	AraC with insertion of LOV2 behind S170
**47**	AraC_T241_LOV2	AraC with insertion of LOV2 behind T241
**48**	AraC_D286_LOV2	AraC with insertion of LOV2 behind D286

**49**	AraC_E3_eYFP	AraC with insertion of eYFP behind E3
**50**	AraC_N16_eYFP	AraC with insertion of eYFP behind N16
**51**	AraC_L23_eYFP	AraC with insertion of eYFP behind L23
**52**	AraC_T50_eYFP	AraC with insertion of eYFP behind T50
**53**	AraC_Q60_eYFP	AraC with insertion of eYFP behind Q60
**54**	AraC_I113_eYFP	AraC with insertion of eYFP behind I113
**55**	AraC_N116_eYFP	AraC with insertion of eYFP behind N116
**56**	AraC_E165_eYFP	AraC with insertion of eYFP behind E165
**57**	AraC_S170_eYFP	AraC with insertion of eYFP behind S170
**58**	AraC_T241_eYFP	AraC with insertion of eYFP behind T241
**59**	AraC_E3_ERD	AraC with insertion of ERD behind E3
**60**	AraC_N16_ERD	AraC with insertion of ERD behind N16
**61**	AraC_L23_ERD	AraC with insertion of ERD behind L23
**62**	AraC_T50_ERD	AraC with insertion of ERD behind T50
**63**	AraC_Q60_ERD	AraC with insertion of ERD behind Q60
**64**	AraC_I113_ERD	AraC with insertion of ERD behind I113
**65**	AraC_N116_ERD	AraC with insertion of ERD behind N116
**66**	AraC_E165_ERD	AraC with insertion of ERD behind E165
**67**	AraC_S170_ERD	AraC with insertion of ERD behind S170
**68**	AraC_T241_ERD	AraC with insertion of ERD behind T241
**69**	AraC_E3_uniRapR	AraC with insertion of uniRapR behind E3
**70**	AraC_N16_uniRapR	AraC with insertion of uniRapR behind N16
**71**	AraC_L23_uniRapR	AraC with insertion of uniRapR behind L23
**72**	AraC_T50_uniRapR	AraC with insertion of uniRapR behind T50
**73**	AraC_Q60_uniRapR	AraC with insertion of uniRapR behind Q60
**74**	AraC_I113_uniRapR	AraC with insertion of uniRapR behind I113
**75**	AraC_N116_uniRapR	AraC with insertion of uniRapR behind N116
**76**	AraC_E165_uniRapR	AraC with insertion of uniRapR behind E165
**77**	AraC_S170_uniRapR	AraC with insertion of uniRapR behind S170
**78**	AraC_T241_uniRapR	AraC with insertion of uniRapR behind T241
**79**	TVMV_L5_PDZ	TVMV with insertion of PDZ behind L5
**80**	TVMV_D11_PDZ	TVMV with insertion of PDZ behind D11
**81**	TVMV_G37_PDZ	TVMV with insertion of PDZ behind G37
**82**	TVMV_I42_PDZ	TVMV with insertion of PDZ behind I42
**83**	TVMV_L56_PDZ	TVMV with insertion of PDZ behind L56
**84**	TVMV_T105_PDZ	TVMV with insertion of PDZ behind T105
**85**	TVMV_S121_PDZ	TVMV with insertion of PDZ behind S121
**86**	TVMV_H143_PDZ	TVMV with insertion of PDZ behind H143
**87**	TVMV_F187_PDZ	TVMV with insertion of PDZ behind F187
**88**	TVMV_D193_PDZ	TVMV with insertion of PDZ behind D193
**89**	TVMV_W198_PDZ	TVMV with insertion of PDZ behind W198
**90**	TVMV_F204_PDZ	TVMV with insertion of PDZ behind F204
**91**	TVMV_I209_PDZ	TVMV with insertion of PDZ behind I209
**92**	Flp_L15_PDZ	Flp with insertion of PDZ behind L15
**93**	Flp_C42_PDZ	Flp with insertion of PDZ behind C42
**94**	Flp_D115_PDZ	Flp with insertion of PDZ behind D115
**95**	Flp_S129_PDZ	Flp with insertion of PDZ behind S129
**96**	Flp_L151_PDZ	Flp with insertion of PDZ behind L151
**97**	Flp_I239_PDZ	Flp with insertion of PDZ behind I239
**98**	Flp_N290_PDZ	Flp with insertion of PDZ behind N290
**99**	Flp_W330_PDZ	Flp with insertion of PDZ behind W330
**100**	Flp_S397_PDZ	Flp with insertion of PDZ behind S397

**101**	Flp_Y403_PDZ	Flp with insertion of PDZ behind Y403
**102**	AraC_E3I	AraC with E3I mutation
**103**	AraC_A28Y	AraC with A28Y mutation
**104**	AraC_T50S	AraC with T50S mutation
**105**	AraC_I113D	AraC with I113D mutation
**106**	AraC_G141D	AraC with G141D mutation
**107**	AraC_G141V	AraC with G141V mutation
**108**	AraC_G141Y	AraC with G141Y mutation
**109**	AraC_E165I	AraC with E165I mutation
**110**	AraC_T241C	AraC with T241C mutation
**111**	AraC_V284F	AraC with V284F mutation
**112**	AraC_V284I	AraC with V284I mutation
**113**	AraC_I113_LOV_E3I	AraC_I113_LOV with E3I mutation
**114**	AraC_I113_LOV_A28Y	AraC_I113_LOV with A28Y mutation
**115**	AraC_I113_LOV_T50S	AraC_I113_LOV with T50S mutation
**116**	AraC_I113_LOV_G141D	AraC_I113_LOV with G141D mutation
**117**	AraC_I113_LOV_G141V	AraC_I113_LOV with G141V mutation
**118**	AraC_I113_LOV_G141Y	AraC_I113_LOV with G141Y mutation
**119**	AraC_I113_LOV_E165I	AraC_I113_LOV with E165I mutation
**120**	AraC_I113_LOV_T241C	AraC_I113_LOV with T241C mutation
**121**	AraC_I113_LOV_V284F	AraC_I113_LOV with V284F mutation
**122**	AraC_I113_LOV_V284I	AraC_I113_LOV with V284I mutation
**123**	AraC_S170_LOV_E3I	AraC_S170_LOV with E3I mutation
**124**	AraC_S170_LOV_A28Y	AraC_S170_LOV with A28Y mutation
**125**	AraC_S170_LOV_T50S	AraC_S170_LOV with T50S mutation
**126**	AraC_S170_LOV_I113D	AraC_S170_LOV with I113D mutation
**127**	AraC_S170_LOV_G141D	AraC_S170_LOV with G141D mutation
**128**	AraC_S170_LOV_G141V	AraC_S170_LOV with G141V mutation
**129**	AraC_S170_LOV_G141Y	AraC_S170_LOV with G141Y mutation
**130**	AraC_S170_LOV_T241C	AraC_S170_LOV with T241C mutation
**131**	AraC_S170_LOV_V284F	AraC_S170_LOV with V284F mutation
**132**	AraC_S170_LOV_V284I	AraC_S170_LOV with V284I mutation

Table S4. Amino acid sequences of the used proteins and insert domains. Tags linked to the proteins are marked in bold.

Protein	Sequence
AraC	MSAEAQNDPLLPGYSFNAHLVAGLTPIEANGYLDFFIDRPLGMKGYILNLTIRGQGVVKNQGR<br>EFVCRPGDILLFPPGEIHHYGRHPEAREWYHQWVYFRPRAYWHEWLNWPSIFANTGFFRPDEA<br>HQPHFSDLFGQIINAGQGEGRYSELLAINLLEQLLLRRMEAINESLHPPMDNRVREACQYISD<br>HLADSNFDIASVAQHVCLSPSRLSHLFRQQLGISVLSWREDQRISQAKLLLSTTRMPIATVGR<br>NVGFDDQLYFSRVFKKCTGASPSEFRAGCEEKVNDVAVKLSGHHHHHH
*As*LOV2	LATTLERIEKNFVITDPRLPDNPIIFASDSFLQLTEYSREEILGRNCRFLQGPETDRATVRKI<br>RDAIDNQTEVTVQLINYTKSGKKFWNLFHLQPMRDQKGDVQYFIGVQLDGTEHVRDAAEREGV<br>MLIKKTAENIDEAAK
ER domain	GPLDNSLALSLTADQMVSALLDAEPPILYSEYDPTRPFSEASMMGLLTNLADRELVHMINWAK<br>RVPGFVDLTLHDQVHLLECAWLEILMIGLVWRSMEHPGKLLFAPNLLLDRNQGKCVEGMVEIF<br>DMLLATSSRFRMMNLQGEEFVCLKSIILLNSGVYTFLSSTLKSLEEKDHIHRVLDKITDTLIH<br>LMAKAGLTLQQQHQRLAQLLLILSHIRHMSNKGMEHLYSMKCKNVVPLYDLLLEMLDAHRLHA<br>PGSEL
eYFP	VSKGEELFTGVVPILVELDGDVNGHKFSVSGEGEGDATYGKLTLKFICTTGKLPVPWPTLVTT<br>FGYGLQCFARYPDHMKQHDFFKSAMPEGYVQERTIFFKDDGNYKTRAEVKFEGDTLVNRIELK<br>GIDFKEDGNILGHKLEYNYNSHNVYIMADKQKNGIKVNFKIRHNIEDGSVQLADHYQQNTPIG<br>DGPVLLPDNHYLSYQSKLSKDPNEKRDHMVLLEFVTAAGITLGMDELYK
Flp recombinase	MSPQFGILCKTPPKVLVRQFVERFERPSGEKIALCAAELTYLCWMITHNGTAIKRATFMSYNT<br>IISNSLSFDIVNKSLQFKYKTQKATILEASLKKLIPAWEFTIIPYYGQKHQSDITDIVSSLQL<br>QFESSEEADKGNSHSKKMLKALLSEGESIWEITEKILNSFEYTSRFTKTKTLYQFLFLATFIN<br>CGRFSDIKNVDPKSFKLVQNKYLGVIIQCLVTETKTSVSRHIYFFSARGRIDPLVYLDEFLRN<br>SEPVLKRVNRTGNSSSNKQEYQLLKDNLVRSYNKALKKNAPYSIFAIKNGPKSHIGRHLMTSF<br>LSMKGLTELTNVVGNWSDKRASAVARTTYTHQITAIPDHYFALVSRYYAYDPISKEMIALKDE<br>TNPIEEWQHIEQLKGSAEGSIRYPAWNGIISQEVLDYLSSYINRRISGHHHHHH
mRFP1	MASSEDVIKEFMRFKVRMEGSVNGHEFEIEGEGEGRPYEGTQTAKLKVTKGGPLPFAWDILSP<br>QFQYGSKAYVKHPADIPDYLKLSFPEGFKWERVMNFEDGGVVTVTQDSSLQDGEFIYKVKLRG<br>TNFPSDGPVMQKKTMGWEASTERMYPEDGALKGEIKMRLKLKDGGHYDAEVKTTYMAKKPVQL<br>PGAYKTDIKLDITSHNEDYTIVEQYERAEGRHSTGA
PDZ domain	RRRVTVRKADAGGLGISIKGGRENKMPILISKIFKGLAADQTEALFVGDAILSVNGEDLSSAT<br>HDEAVQALKKTGKEVVLEVKYMK
SigF	MSDVEVKKNGKNAQLKDHEVKELIKQSQNGDQQARDLLIEKNMRLVWSVVQRFLNRGYEPDDL<br>FQIGCIGLLKSVDKFDLTYDVRFSTYAVPMIIGEIQRFIRDDGTVKVSRSLKELGNKIRRAKD<br>ELSKTLGRVPTVQEIADHLEIEAEDVVLAQEAVRAPSSIHETVYENDGDPITLLDQIADNSEE<br>KWFDKIALKEAISDLEEREKLIVYLRYYKDQTQSEVAERLGISQVQVSRLEKKILKQIKVQMD<br>HTDG
TVMV protease	MSSKALLKGVRDFNPISACVCLLENSSDGHSERLFGIGFGPYIIANQHLFRRNNGELTIKTMH<br>GEFKVKNSTQLQMKPVEGRDIIVIKMAKDFPPFPQKLKFRQPTIKDRVCMVSTNFQQKSVSSL<br>VSESSHIVHKEDTSFWQHWITTKDGQCGSPLVSIIDGNILGIHSLTHTTNGSNYFVEFPEKFV<br>ATYLDAADGWCKNWKFNADKISWGSFTLVE
uniRapR	TCVVHYTGMLEDGKKFDSSRDRNKPFKFMLGKQEVIRGWEEGVAQMSVGQRAKLTISPDYAYG<br>ATGHGSGSGSGVKDLLQAWDLYYHVFRRISGPPGPGSGLWHEMWHEGLEEASRLYFGERNVKG<br>MFEVLEPLHAMMERGPQTLKETSFNQAYGRDLMEAQEWCRKYMKSGSSGGSGSGIIPPHATLV<br>FDVELLKLE

Table S5. PDB IDs of protein structures shown and used in this study.

Structure	PDB-ID	Reference
AraC, apo-form	2ARA	[21]
AraC, complexed with L-arabinose	2ARC	[21]
AraC, DBD	2K9S	[22]
*As*LOV2 domain	2V0W, 2V0U	[23]
ERD	1A52	[24]
eYFP, F165G	6ZQO	[25]
Flp recombinase	1FLO	[26]
PDZ	1Z86	[27]
Rob transcription factor	1D5Y	[28]
TVMV protease	3MMG	[29]
uniRapR	1FAP	[30]

Supplementary References

[1] M. Akdel, D. E. V. Pires, E. P. Pardo, J. Jänes, A. O. Zalevsky, B. Mészáros, P. Bryant, L. L. Good, R. A. Laskowski, G. Pozzati, A. Shenoy, W. Zhu, P. Kundrotas, V. R. Serra, C. H. M. Rodrigues, A. S. Dunham, D. Burke, N. Borkakoti, S. Velankar, A. Frost, J. Basquin, K. Lindorff-Larsen, A. Bateman, A. V. Kajava, A. Valencia, S. Ovchinnikov, J. Durairaj, D. B. Ascher, J. M. Thornton, N. E. Davey, A. Stein, A. Elofsson, T. I. Croll, P. Beltrao, *Nat Struct Mol Biol* **2022**, *29*, 1056.

[2] T. Saldaño, N. Escobedo, J. Marchetti, D. J. Zea, J. Mac Donagh, A. J. Velez Rueda, E. Gonik, A. García Melani, J. Novomisky Nechcoff, M. N. Salas, T. Peters, N. Demitroff, S. Fernandez Alberti, N. Palopoli, M. S. Fornasari, G. Parisi, *Bioinform* **2022**, *38*, 2742.

[3] C. J. Wilson, W.-Y. Choy, M. Karttunen, *Int J Mol Sci* **2022**, *23*, 4591.

[4] J. Jumper, R. Evans, A. Pritzel, T. Green, M. Figurnov, O. Ronneberger, K. Tunyasuvunakool, R. Bates, A. Žídek, A. Potapenko, A. Bridgland, C. Meyer, S. A. A. Kohl, A. J. Ballard, A. Cowie, B. Romera-Paredes, S. Nikolov, R. Jain, J. Adler, T. Back, S. Petersen, D. Reiman, E. Clancy, M. Zielinski, M. Steinegger, M. Pacholska, T. Berghammer, S. Bodenstein, D. Silver, O. Vinyals, A. W. Senior, K. Kavukcuoglu, P. Kohli, D. Hassabis, *Nature* **2021**, *596*, 583.

[5] A. A. K. Nielsen, B. S. Der, J. Shin, P. Vaidyanathan, V. Paralanov, E. A. Strychalski, D. Ross, D. Densmore, C. A. Voigt, *Science* **2016**, *352*, DOI 10.1126/science.aac7341.

[6] A. A. Green, J. Kim, D. Ma, P. A. Silver, J. J. Collins, P. Yin, *Nature* **2017**, *548*, 117.

[7] Z. Chen, R. D. Kibler, A. Hunt, F. Busch, J. Pearl, M. Jia, Z. L. VanAernum, B. I. M. Wicky, G. Dods, H. Liao, M. S. Wilken, C. Ciarlo, S. Green, H. El-Samad, J. Stamatoyannopoulos, V. H. Wysocki, M. C. Jewett, S. E. Boyken, D. Baker, *Science* **2020**, *368*, 78.

[8] X. J. Gao, L. S. Chong, M. S. Kim, M. B. Elowitz, *Science* **2018**, *361*, 1252.

[9] Z. Chen, J. M. Linton, R. Zhu, M. B. Elowitz, *bioRxiv*, **2022**.

[10] Y. L. Vishweshwaraiah, J. Chen, V. R. Chirasani, E. D. Tabdanov, N. V. Dokholyan, *Nat Commun* **2021**, *12*, 6615.

[11] J. Dietler, R. Schubert, T. G. A. Krafft, S. Meiler, S. Kainrath, F. Richter, K. Schweimer, M. Weyand, H. Janovjak, A. Möglich, *J Mol Biol* **2021**, *433*, 167107.

[12] F. Ceroni, A. Boo, S. Furini, T. E. Gorochowski, O. Borkowski, Y. N. Ladak, A. R. Awan, C. Gilbert, G.-B. Stan, T. Ellis, *Nat Methods* **2018**, *15*, 387.

[13] A. Eldar, M. B. Elowitz, *Nature* **2010**, *467*, 167.

[14] M. Suyama, O. Ohara, *Bioinformatics* **2003**, *19*, 673.

[15] R. A. George, J. Heringa, *Protein Eng Des Sel* **2002**, *15*, 871.

[16] K. Bae, B. K. Mallick, C. G. Elsik, *Bioinform* **2005**, *21*, 2264.

[17] M. Prabhakaran, *Biochem J* **1990**, *269*, 691.

[18] R. Bhaskaran, P. k. Ponnuswamy, *Int J of Pept Protein Res* **1988**, *32*, 241.

[19] K. Takano, K. Yutani, *J Mol Biol,* **1998**, 280, 749.

[20] H. Zhou, Y. Zhou, *Proteins* **2004**, *54*, 315.

[21] S. M. Soisson, B. MacDougall-Shackleton, R. Schleif, C. Wolberger, *Science* **1997**, *276*, 421.

[22] M. E. Rodgers, R. Schleif, *Proteins* **2009**, *77*, 202.
[23] A. S. Halavaty, K. Moffat, *Biochemistry* **2007**, *46*, 14001.
[24] D. M. Tanenbaum, Y. Wang, S. P. Williams, P. B. Sigler, *Proc Natl Acad Sci USA* **1998**, *95*, 5998.
[25] N. V. Pletneva, E. G. Maksimov, E. A. Protasova, A. V. Mamontova, T. R. Simonyan, R. H. Ziganshin, K. A. Lukyanov, L. Muslinkina, S. Pletnev, A. M. Bogdanov, V. Z. Pletnev, *Computational and Structural Biotechnol J* **2021**, *19*, 2950.
[26] Y. Chen, U. Narendra, L. E. Iype, M. M. Cox, P. A. Rice, *Mol Cell* **2000**, *6*, 885.
[27] J. Yan, W. Wen, W. Xu, J. Long, M. E. Adams, S. C. Froehner, M. Zhang, *EMBO J* **2005**, *24*, 3985.
[28] H. J. Kwon, M. H. J. Bennik, B. Demple, T. Ellenberger, *Nat Struct Mol Biol* **2000**, *7*, 424.
[29] P. Sun, B. P. Austin, J. Tözsér, D. S. Waugh, *Protein Sci* **2010**, *19*, 2240.
[30] J. Choi, J. Chen, S. L. Schreiber, J. Clardy, *Science* **1996**, *273*, 239.
